# Full-thickness skin grafts in nasal reconstruction: A retrospective study

**DOI:** 10.1016/j.jdin.2023.08.004

**Published:** 2023-08-12

**Authors:** Joyce Chen, Collin M. Costello, Carolyn Mead-Harvey, Katie L. Kunze, Juan-Carlos Martinez, Shari A. Ochoa

**Affiliations:** a Department of Internal Medicine, California Pacific Medical Center, San Fransisco, California; b Department of Dermatology, Mayo Clinic, Scottsdale, Arizona; c Department of Quantitative Health Sciences, Mayo Clinic, Scottsdale, Arizona; d Advanced Dermatology and Cosmetic Surgery, Jacksonville, Florida

**Keywords:** full-thickness skin grafts, Mohs micrographic surgery, nasal reconstruction, nasal surgery, reconstructive surgical procedure, skin transplantation

*To the Editor:* The nose is the most common site for skin cancer on the head and neck.^1^ In appropriately selected patients, utilizing an optimal donor site, full-thickness skin grafts (FTSGs) can provide excellent functional and cosmetic outcomes.^2^ This study reports our experience with FTSG to repair nasal defects.

We conducted a retrospective chart review from 2008 to 2018 at our institution’s Arizona and Florida campuses. Cases were identified using the billing codes 15260 plus 17311. Inclusion criteria included Mohs micrographic surgery performed on the nose with same day repair with FTSG. A total of 544 cases were identified. Surgical complications were defined as hematoma, partial or complete graft failure, donor-site infection, and FTSG-site infection.

Patient demographics and tumor and defect characteristics are summarized in Table I. A total of 21 postoperative complications were recorded (3.9%). Of these, 5 were due to partial (4 cases, 0.7%) or complete graft failure (1 case, 0.2%). There were 2 postoperative hematomas (0.4%). Postoperative infection of the FTSG site occurred in 3 cases (0.6%), and postoperative infection of the donor site developed in 11 cases (2.0%). In 253 cases (47%), a bolster was used to secure the graft after placement, and it was not used in 287 cases (53%). Eight patients (2.8%) without the bolster experienced a nondonor–site complication (postoperative infection, postoperative hematoma, graft failure), compared to 2 (0.8%) patients with bolster (95% CI: −0.4, 4.8). No complications were seen in transplant patients or smokers.

**Table I:** Patient characteristics and surgery outcomes by complication status

	All patients (*n* = 542)	No complication (*n* = 52)	Complication (*n* = 21)	Difference (95% CI)
Gender				
Male	258 (47%)	247 (47%)	11 (52.4%)	−5.1% (−27.5, 18.4)
Female	284 (52%)	274 (52.7%)	10 (47.6%)	4.9% (−18.6, 27.1)
Age, y				
Mean (SD)	72.9 (13.19)	73.0 (13.11)	70.2 (15.10)	2.78 (−2.98, 8.55)
Race				
White	529 (99.6%)	509 (99.6%)	20 (100.0%)	−0.4% (−2.1, 20.4)
Anticoagulation status				
No	239 (44.7%)	229 (44.5%)	10 (50.0%)	−5.5% (−28.8, 18.1)
Yes	296 (55.3%)	286 (55.5%)	10 (50.0%)	5.5% (−18.1, 28.8)
Smoking Status				
No	498 (94.9%)	477 (94.6%)	21 (100.0%)	−5.4% (−8.3, 15.2)
Yes	27 (5.1%)	27 (5.4%)	0 (0.0%)	
Transplant patient				
No	515 (96.6%)	494 (96.5%)	21 (100.0%)	−3.5% (−6.2, 17.0)
Yes	18 (3.4%)	18 (3.5%)	0 (0.0%)	
Tumor				
BCC	470 (86.7%)	452 (86.8%)	18 (85.7%)	1.0% (−10.1, 25.4)
SCC	49 (9.0%)	48 (9.2%)	1 (4.8%)	
MM/MIS	24 (4.4%)	16 (3.1%)	1 (4.8%)	
Nasal subunit				
Root	12 (2.2%)	12 (2.3%)	0 (0.0%)	
Dorsum	56 (10.3%)	55 (10.5%)	1 (4.8%)	5.8% (−16.6, 11.7)
Lateral side Wall	42 (7.7%)	42 (8.0%)	0 (0.0%)	
Tip	199 (36.6%)	191 (36.6%)	8 (38.1%)	−1.5% (−25.9, 18.7)
Ala nasi	230 (42.4%)	217 (41.6%)	13 (61.9%)	−20.3% (−40.3, 5.8)
Soft triangle	3 (0.6%)	3 (0.6%)	0 (0.0%)	
Columella	3 (0.6%)	3 (0.6%)	0 (0.0%)	
Donor site				
Preauricular	152 (28.2%)	149 (28.8%)	3 (14.3%)	14.5% (−11.0, 26.0)
Postauricular	79 (14.7%)	76 (14.7%)	3 (14.3%)	0.4% (−24.0, 11.6)
Conchal bowl	111 (20.6%)	100 (19.3%)	11 (52.4%)	−33.1% (−55.1, −8.2)
Nasolabial fold	14 (2.6%)	14 (2.7%)	0 (0.0%)	
Supraclavicular	18 (3.3%)	17 (3.3%)	1 (4.8%)	−1.5% (−23.4, 3.7)
Infraclavicular	6 (1.1%)	6 (1.2%)	0 (0.0%)	
Burow’s graft nasal	105 (19.5%)	102 (19.7%)	3 (14.3%)	5.4% (−19.2, 16.7)
Other Burow’s	39 (7.2%)	39 (7.5%)	0 (0.0%)	
Forehead	14 (2.6%)	14 (2.7%)	0 (0.0%)	
Upper eyelid	1 (0.2%)	1 (0.2%)	0 (0.0%)	
Postoperative diameter (cm)				
Mean (SD)	1.4 (0.74)	1.4 (0.74)	1.3 (0.67)	0.07 (−0.26, 0.40)
Graft surface area (cm^2^)				
Mean (SD)	1.9 (2.42)	1.9 (2.45)	1.6 (1.46)	0.30 (−0.79, 1.38)
Bolster				
Yes	25 (46.9%)	250 (48.2%)	3 (14.3%)	33.9% (−0.1, 45.5)
No	287 (53.1%)	269 (51.8%)	18 (85.7%)	−33.9% (−45.5, 0.1)
Postsurgery scar refinement				
Dermabrasion	11 (2.0%)	11 (2.1%)	0 (0.0%)	
Laser	11 (2.0%)	10 (1.9%)	1 (4.8%)	−2.8% (−24.8, 2.1)
Kenalog	10 (1.8%)	9 (1.7%)	1 (4.8%)	−3.0% (−25.1, 1.9)

%, Percentage; *BCC*, basal cell carcinoma; *cm*, centimeter; *MIS*, melanoma in situ; *MM*, malignant melanoma; *n*, number; *SCC*, squamous cell carcinoma.

This study presents the postoperative results of 542 patients who underwent the FTSG repair to reconstruct nasal defects, demonstrating that FTSGs represent a viable, cosmetically sound reconstruction option regardless of the nasal subunit (Fig 1). In our study, patients who underwent nasal FTSG experienced a very low postoperative complication rate (3.9%), compared to previous literature reports ranging from 11.7 to 18.3%.^3^^,^^4^ There was no significant difference in donor site, postoperative defect diameter, and graft surface area between cases with and without complications. Of the 10 patients who experienced postoperative complications of the graft site, 8 did not have a bolster dressing applied to their graft. Although the number of complications in our study was too low to have power to detect statistically significant differences, it suggests that bolster dressings may assist with graft take^5^ and play a protective role in preventing graft-site complications. In addition, complication rates were not increased for transplant patients, active smokers, or patients on systemic anticoagulation. This suggests that FTSG can be safely considered in patients with risk factors for surgical complications, although we acknowledge that the limited power of our study may not fully highlight the consequences of these risk factors. Of note, patients on systemic anticoagulation were routinely counseled to continue anticoagulation, whereas counseling regarding smoking cessation was more variable and patient/surgeon dependent. Lastly, use of FTSG allowed for acceptable aesthetic outcomes, with only 5.7% of patients requiring a form of scar refinement.

**Fig 1:**
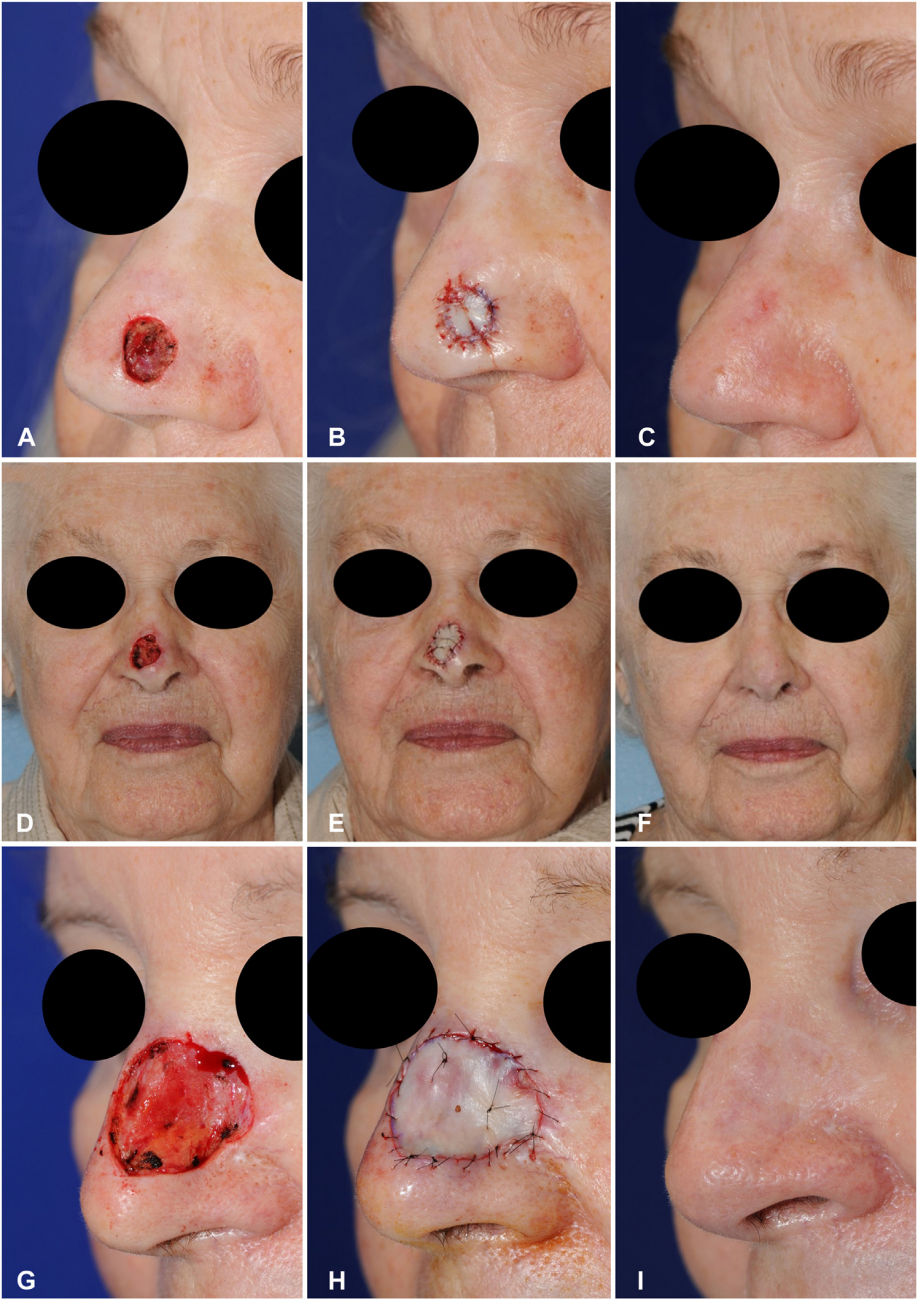
Full-thickness skin graft reconstructions. **A**, Initial defect. **B**, After graft placement. **C**, Four months after reconstruction. **D**, Initial defect. **E**, After graft placement. **F**, Four months after reconstruction. **G**, Initial defect. **H**, After graft placement. **I**, Four months after reconstruction.

In conclusion, our findings show that FTSGs are a versatile, safe, and aesthetically sound option for single-staged reconstruction of nasal defects. Additionally, the use of bolsters may decrease the likelihood of a postoperative complication.

## Conflicts of interest

None disclosed.
